# Effects of Strength Training Combined with Specific Plyometric exercises on body composition, vertical jump height and lower limb strength development in elite male handball players: a case study

**DOI:** 10.2478/hukin-2014-0040

**Published:** 2014-07-08

**Authors:** Alberto Carvalho, Paulo Mourão, Eduardo Abade

**Affiliations:** 1 Research Center in Sports Science, Health and Human Development (CIDESD), Portugal.; 2 Human Movement Laboratory – Higher Education Institute of Maia (ISMAI), Portugal.

**Keywords:** strength training, specific plyometric exercises, vertical jump, handball

## Abstract

The purpose of the present study was to identify the effects of a strength training program combined with specific plyometric exercises on body composition, vertical jump (VJ) height and strength development of lower limbs in elite male handball players. A 12-week program with combined strength and specific plyometric exercises was carried out for 7 weeks. Twelve elite male handball players (age: 21.6 ± 1.73) competing in the Portuguese Major League participated in the study. Besides the anthropometric measurements, several standardized jump tests were applied to assess VJ performance together with the strength development of the lower limbs in an isokinetic setting. No significant changes were found in body circumferences and diameters. Body fat content and fat mass decreased by 16.4 and 15.7% respectively, while lean body mass increased by 2.1%. Despite small significance, there was in fact an increase in squat jump (SJ), counter movement jump (CMJ) and 40 consecutive jumps after the training period (6.1, 3.8 and 6.8%, respectively). After the applied protocol, peak torque increased in lower limb extension and flexion in the majority of the movements assessed at 90ºs-1. Consequently, it is possible to conclude that combining general strength-training with plyometric exercises can not only increase lower limb strength and improve VJ performance but also reduce body fat content.

## Introduction

Much has been written to support that muscle fibres respond to resistance training with an increase in the cross sectional area and force generating capacity (Moore et al., 2004). For instance, hypertrophic and neural adaptations are commonly used to induce structural changes in muscle morphology and increase the rate of force development (Verkhoshansky, 2006). Additionally, power and plyometric exercises are important to develop the muscle stretch shortening cycle capacity (Komi, 2000). In essence, strength training adaptations contribute to the increase of the muscular power output, the development of the capacity of energy producing systems of the body and the ability to improve motor power potential in competition (Verkhoshansky, 2006).

Handball is characterized by high intensity actions performed at high velocities (Rogulj et al., 2004); therefore, success depends partly on well-developed muscular strength. Even if handball training is able to reproduce most of the patterns required for the conditioning of the players (Impellizzeri et al., 2006), the replication of the muscle demands seems much more difficult. Thus, it is necessary to use intense and specific external loads to benefit from considerable muscular adaptations, which is only possible with specific strength training sessions (Zatsiorsky and Kraemer, 2006). Accordingly, knowledge about strength training planning is decisive when increasing strength endurance, coordination, explosive strength, speed and technical abilities (Issurin, 2010).

Understanding of the effects of specific strength and plyometric training programs on body composition, VJ performance and muscular strength can help coaches select the best training stimulus in order to improve the individual performance of their athletes. The majority of specific handball actions involve stretch shortening cycles, based on the fact that a concentric action produces higher peak torque when preceded by an eccentric contraction (Enoka, 1997; Hakkinen et al., 2000; Komi, 2000). As a result, strength training is essential to develop VJ capacity (Marques and Gonzalez-Badillo, 2006) and, consequently, to perform specific offensive (jump shots) and defensive (blocks) handball motor activities.

VJ performance has been related to maximal force, the rate of force development and stretch-shortening cycle capacity (Rimmer and Sleivert, 2000). Therefore, VJ assessment is a useful index of the muscular ability to generate power and can be used to monitor a performance level (Quagliarella et al., 2010). Previous studies have shown improvements in jumping performance after several training sessions with depth jumps (Bobbert, 1990), additional loads (Wilson et al., 1993), combined strength and plyometric training (Adams et al., 1992), only plyometric training (Humphries et al., 1995) and combined electro stimulation and plyometric exercises (Maffiuletti et al., 2002).

To our knowledge, there are very few studies focused on the effects of combining strength training and plyometric exercises. Therefore, the aim of the present study was to identify the effects of a general strength-training program that included plyometric exercises on: i) VJ height in different types of jumps and maximum strength development of lower limbs; ii) body composition changes in elite male handball players.

## Material and Methods

### Subjects

Twelve semi-professional players from the Portuguese Handball Major League (age 21.6 ± 1.73 years; body height 183.9 ± 0.09 cm; body mass 81.7 ± 8.3 kg) participated in this study. All players belonged to a team that had five training units per week. They were also informed about protocol procedures, benefits and risks.

The protocol was constructed in accordance with the Declaration of Helsinki and approved by the Ethics Committee of the Research Center in Sport, Health and Human Development (Portugal).

### Procedures

The assessment of anthropometric and strength parameters was carried out twice: one week before the beginning of the protocol (pre-test) and one week after the training program’s last session (post-test). The anthropometric assessment was based on the calculation of body mass, body height, skinfold thickness (triceps, biceps, subscapular, suprailiac, abdominal, thigh, iliac and calf), body circumference (arm relaxed/ tense, thigh and calf) and body diameter (condylar humeral and femoral). Body fat content was calculated with the Faulkner formula (Faulkner, 1968), and fat and lean body mass by the Siri equation (Siri, 1961).

Maximum dynamic and isometric strength (peak torque) were evaluated by using the isokinetic dynamometer REV 9000 (Technogym, Italy). These isokinetic sessions were performed on different days of the VJ protocol and were always preceded by a general activation of low intensity running and stretching (Mero et al., 2009). The maximum dynamic strength assessment was performed at an angular speed of 90ºs^−1^, with the subject seated and the trunk in a static position. The dynamometer axis was aligned with the lateral knee epicondyle. The knee range of motion was 70º (20 to 90º of flexion). The lever arm was positioned at the distal third of the leg. Torque was gravity-corrected. Dynamometer calibration was performed before every session in accordance with the manufacturer’s instructions. The evaluation protocol consisted of performing five knee extension and flexion actions at 90ºs^−1^ angular velocities, with one minute rest periods between sets. The isometric evaluation was performed during 5 s with the static lever arm. All subjects benefited from visual feedback and verbal encouragement from the same physician. The highest peak torque of the five repetitions was selected for further analysis.

To evaluate VJ performance, the subjects performed three repetitions for each type of the jump with the best score being registered. A 20 s rest period between trials was allowed. Both take-off and landing were performed with both feet, with no initial steps or shuffling. In the SJ, the athlete stood with both feet on a mat with weight evenly distributed. Hands were placed on hips and, after a 90º knee flexion with the trunk straight, the player jumped vertically as high as possible. For CMJs, hands were also placed on the hips and remained so throughout the jump. The athlete performed a 90º knee flexion and immediately jumped vertically, landing back on the mat with both feet at the same time. The 40 consecutive jumps were performed similarly to the CMJ, however, players were required to perform them on the mat with no rest between them. Jump height was calculated with an ergo jump (1000 DigiTimes, Digitest Finland) in accordance with the Bosco Protocol (Bosco et al., 1983).

After the pre-test, a combined 12-week strength and plyometric program was applied (Figure 1). The methodology used was in accordance with the available specific strength training literature (Komi, 2000; Zatsiorsky and Kraemer, 2006).

In the first three weeks of training, resistance exercises were emphasised. Training sessions consisted of 15 exercises focused on both upper and lower limbs. Every exercise was performed in 2 sets of 15–20 repetitions (60 to 75% of 1 RM), an interval of 45 s between exercises and 2 min between sets. The main goal was to learn the exercise motions in order to prevent injuries and prepare athletes for a further load increase with individual load assignments. The exercises included: 1) leg press; 2) leg extension; 3) leg flexion; 4) standing calf raise; 5) cable bent-over triceps extension; 6) lat pull; 7) butterfly; 8) bench press; 9) barbell upright row; 10) sit-up in inclined bench; 11) lower back extensions; 12) cufflink flexors; 13) shoulder external rotation; 14) barbell half squats; 15) reverse and forward lunges with a bar.

The phase 2 involved a hypertrophy training program which lasted 4 weeks with 3 training units per week. The program consisted of 8 exercises (leg press, leg extension, leg flexion, calf muscles, triceps, lat pull, butterfly and bench press) which were conducted in two sets of 8–12 maximal repetitions (75 to 80% of 1 RM) with a 60 s interval between exercises and 2 min between sets. The load increased in all exercises each time a subject performed over 12 RM. The main goal was to increase the muscle cross sectional area.

The third phase involved 3 training units per week focused on neural adaptations and lasted five weeks. The same 8 exercises were performed, but the load was increased. Players performed a pyramid model (6-4-2 maximum repetitions) with a load increasing throughout the sets. The resting intervals were 2 min between exercises and 3.5 min between sets. The load was increased in all exercises each time a subject performed over 6 RM. The main goal of this particular phase was to increase neural adaptations.

In the last seven weeks, a specific plyometric program was added to the strength training program. The plyometric training was based on the combination of concentric and eccentric actions with a similar speed and muscular contraction used in handball specific technical abilities. Upper limbs, trunk and head movements contributed to an increase in jumping performance so as to potentiate the concentric action (positive jump phase). To our understanding, these factors are very important when offensive (jump shots) and defensive (blocks) handball specific actions are performed. Four exercises of 3 sets of 12 reps were used in this plyometric program with no additional loads. An interval of 30 to 40 s of rest was allowed between exercises and 3 min between sets. The plyometric exercises included hurdle jumps, lateral multi-jumps for plantar flexors and leg extensors and frontal multi-jumps. The plyometric program was integrated in the technical training of regular handball practice, 3 times per week. The execution speed was high so as to increase the stretch shortening cycle force development.

### Statistics

A mean and standard deviation descriptive statistics in all variables was used to obtain reliable data characterization. A repeated measurement paired-samples t-test was used to assess the training effects within groups. Significance was maintained at the 0.05 level. All statistical analyses were performed by using SPSS software (V. 11.0).

## Results

Despite the body diameters and circumference values, there were no significant changes (p<0.05) between the pre- and post-tests. Table 1 depicts the average values of the eight skinfolds, body fat percentage as well as the fat and lean body mass. Both fat % and fat mass decreased after the training program. These reductions are supported by the decrease of triceps, biceps, thigh and calf skinfolds, however, lean body mass significantly increased.

Table 2 shows the results of the VJ protocol (SJ, CMJ and 40-consecutive jumps). Although there were no significant differences, there was an increase in the VJ height in the three types of jumps.

Table 3 presents the results of the isokinetic assessment which measured isometric and dynamic strength of leg flexors and extensors at a speed of 90º/s. The majority of the strength manifestations achieved gains between the two moments of assessment, however, the most significant differences were found at ISMLANT (11.4%), ECCLAG (9.6%), ISMRANT (8%) and ECCRANT (6.6%).

## Discussion

The aim of this study was to examine the impact of an in-season strength training program combined with plyometric exercises on body composition, VJ performance and maximum strength development of lower limbs in elite male handball players. In general, the results showed a decrease in body fat percentage, an improvement in jumping height and a greater development of maximal peak torque of the lower limbs.

Body fat mass and fat content were reduced by 15.7% and 16.4%, respectively. Previous studies have already shown that resistance training is able to reduce total fat content after a 14-week (Fleck et al., 2006) and 8-week program (Beni, 2012). In fact, the development of muscle tissue after a strength program seems to increase energy consumption (Beni, 2012) which supports the decrease in fat content. The present 12-week program did not influence lean body mass or body circumference. Thus, this might indicate that muscle hypertrophy did not accompany strength gains, as suggested in a previous study (Ozmun et al., 1994).

An increase in jumping performance was reflected by the absolute and relative gains in SJ (2.24cm, 6.1%), CMJ (1.52cm, 3.8%) and 40-consecutive jumps (2.46m, 6.8%). To our knowledge, there is little literature related to the effect of combined strength and plyometric programs in VJ height of handball players. Despite the lack of specific data, some research, mainly in volleyball, can help support our results. In fact, in some studies, increases of 3.8% in CMJ after a 12-week program of resistance and plyometric exercises (Marques et al., 2008) and an increment of 7% in VJ height for starters and nonstarters in women's volleyball after a strength program during off-season training (Fry et al., 1991) have been found. Thus, strength training seems to be an essential tool in developing VJ performance (Luebbers et al., 2003). Resistance training improves the muscle contractile mechanisms, maximal force capacity and the rate of force development (Rimmer and Sleivert, 2000). Additionally, plyometric training is known to potentiate stretch shortening cycles (Komi, 2000) which is crucial when performing some handball specific motor offensive and defensive actions such as jump shots and blocks. Moreover, combining strength and plyometric training is more effective in the increase of peak torque of leg muscles than the gains obtained after a strength program alone (Fatouros et al., 2000; Harris et al., 2000). Subsequently, combining strength and plyometric exercises is more effective in developing strength, VJ capacity and specific handball motor actions.

The isokinetic assessment showed significant developments in the peak torque of lower limbs. The most significant gains were verified in the isometric and eccentric force, mainly in the knee flexors. This improvement can be justified by the fact that eccentric and isometric actions are not performed with such frequency as concentric actions, so they are more susceptible to training stimuli. Moreover, handball is a game characterized by complex movements that require constant mobility of knee extensors so as to achieve high speed actions and quick changes of direction (van den Tillaar and Ettema, 2007). Since the knee flexors are not that much involved, their strength level is commonly lower, what increased the possibility of obtaining greater strength gains after the specific strength program.

The observed increase in maximal strength was in accordance with the results of previous research (Gorostiaga et al., 2004) which found gains of 13% and 9% in leg extensor and flexor muscles, respectively, after a 6-week period of heavy strength training. Other studies have also showed an improvement in the peak torque of thigh muscles and a concentric ratio after a 4-week heavy strength training program.

## Conclusions

The results of this study showed that elite male handball players (Portuguese Major League) can develop both VJ height and maximal strength of the lower limbs after a combined resistance and plyometric training program. This type of conditioning program can also promote significant changes in body composition of highly trained athletes as shown by the decrease in body fat mass found in this study.

## Practical implications

The present findings suggest that handball coaches should use combined strength and plyometric training programs with regularity throughout the competitive season in order to achieve gains in maximal strength. This improvement in the capacity to generate force seems to potentiate the performance of specific handball motor actions such as the VJ. In addition, we suggest that insufficient strength stimulus can impede the development of specific performance in handball and result in performance deterioration.

## Figures and Tables

**Table 1:** Average values and standard deviation (SD) of skinfolds (mm), body fat (%) fat and lean body mass (kg) with the respective absolute (Abs) and percentual (%) gains as well as t and p values at pre and post test.

	Pre test		Post test		Gains		Value	

	Mean	SD	Mean	SD	Abs.	%	t	P
Triceps (mm)	12,41	±5,49	10,06	±4,06	−2,36	−23,4	3,38	0,015^*^
Biceps (mm)	8,34	±3,20	5,87	±1,58	−2,47	−42,1	3,35	0,015^*^
Subscapular (mm)	14,89	±2,71	15,24	±4,04	0,36	2,3	−0,21	0,841
Suprailiac (mm)	9,86	±4,02	6,97	±2,26	−2,89	−41,4	2,22	0,068
Abdominal (mm)	19,24	±6,47	17,14	±6,32	−2,10	−12,3	1,39	0,214
Thigh (mm)	18,77	±4,04	14,70	±5,18	−4,07	−27,7	4,78	0,003^*^
Calf (mm)	12,40	±5,63	9,51	±4,78	−2,89	−30,3	3,87	0,008^*^
Iliac (mm)	12,74	±3,69	16,46	±6,22	3,71	22,6	−2,27	0,063
Body Fat (%)	13,94	±3,19	11,98	±3,28	−1,97	−16,4	2,79	0,032^*^
Fat Mass (Kg)	11,02	±2,61	9,53	±2,99	−1,49	−15,7	2,85	0,029^*^
Lean Mass (Kg)	68,13	±6,08	69,62	±5,35	1,49	2,1	−2,85	0,029^*^

* Significative differences to p <0.05

**Table 2:** Average values and standard deviation (SD) of squat jump, counter movement jump and 40 consecutive jumps, with the respective absolute (Abs) and percentual (%) gains as well as t and p values at pre and post test

	Pre test		Post test		Gains		Value	

	Mean	SD	Mean	SD	Abs.	%	t	P
SJ (cm)	34,56	±7,89	36,80	±6,34	2,24	6,1	−2,085	0,105
CMJ (cm)	38,68	±8,12	40,20	±8,58	1,52	3,8	−1,088	0,338
40 Jumps (cm)	33,46	±7,09	35,92	±5,41	2,46	6,8	−1,876	0,134

* Significative differences to p <0.05

**Table 3:** Average values and standard deviation (SD) of isokinetic protocol, with the respective absolute (Abs) and percentual (%) gains as well as t and p values at pre and post test

N^*^m	Pre test		Pos test		Gains		Value	

	Mean	SD	Mean	SD	Abs.	%	t	P
ISMLAG	286,30	±38,21	292,80	±38,34	6,50	2,2	−0,853	0,416
ISMLANT	120,00	±20,13	135,40	±15,13	15,40	11,4	−2,871	0,018^*^
ECCLAG	320,90	±50,11	355,10	±52,28	34,20	9,6	−6,163	0,000^*^
ECCLANT	170,60	±15,52	179,00	±29,60	8,40	4,7	−1,109	0,296
CONCLAG	246,30	±28,71	249,50	±24,58	3,20	1,3	−0,568	0,584
CONCLAN	152,70	±29,23	159,60	±26,11	6,90	4,3	−1,189	0,265
ISMRAG	295,00	±41,71	302,00	±38,74	7,00	2,3	−0,496	0,633
ISMRANT	137,44	±22,93	149,33	±26,16	11,89	8,0	−2,307	0,050^*^
ECCRAG	345,00	±39,87	361,78	±43,28	16,78	4,6	−1,352	0,213
ECCRANT	178,67	±21,48	191,33	±22,93	12,67	6,6	−2,635	0,030^*^
CONCRAG	258,22	±38,22	257,11	±27,03	−1,11	−0,4	0,246	0,812
CONCRAN	179,11	±35,03	177,33	±18,10	−1,78	−1,0	0,183	0,859

* Significant differences to p < 0,05

ISMLAG – Isometric left agonist; ISMLANT – Isometric left antagonist; ECCLAG – Eccentric left agonist; ECCLANT – Eccentric left antagonist; CONCLAG – Concentric left agonist; CONCLAN – Concentric left antagonist; ISMRAG – Isometric right agonist; ISMRANT – Isometric right antagonist; ECCRAG – Eccentric right agonist; ECCRANT – Eccentric right antagonist; CONCRAG – Concentric right agonist; CONCRAN –Concentric right antagonist
